# A prediction model for massive hemorrhage in trauma: a retrospective observational study

**DOI:** 10.1186/s12873-022-00737-y

**Published:** 2022-11-14

**Authors:** Chengyu Guo, Minghui Gong, Lei Ji, Fei Pan, Hui Han, Chunping Li, Tanshi Li

**Affiliations:** 1 Present Address: School of Medicine, Nankai University, Tianjin, 300071 China; 2 Department of Emergency, First Medical Center, Chinese PLA General Hospital, Beijing, 100853 China; 3 School of Software, Tsinghua University, Beijing, 100084 China; 4 Department of Information, Medical Supplies Center of PLA General Hospital, Beijing, 100853 China

**Keywords:** Trauma, Massive hemorrhage, LASSO, Prediction model, Assisted diagnosis

## Abstract

**Background:**

Massive hemorrhage is the main cause of preventable death after trauma. This study aimed to establish prediction models for early diagnosis of massive hemorrhage in trauma.

**Methods:**

Using the trauma database of Chinese PLA General Hospital, two logistic regression (LR) models were fit to predict the risk of massive hemorrhage in trauma. Sixty-two potential predictive variables, including clinical symptoms, vital signs, laboratory tests, and imaging results, were included in this study. Variable selection was done using the least absolute shrinkage and selection operator (LASSO) method. The first model was constructed based on LASSO feature selection results. The second model was constructed based on the first vital sign recordings of trauma patients after admission. Finally, a web calculator was developed for clinical use.

**Results:**

A total of 2353 patients were included in this study. There were 377 (16.02%) patients with massive hemorrhage. The selected predictive variables were heart rate (OR: 1.01; 95% CI: 1.01–1.02; *P*<0.001), pulse pressure (OR: 0.99; 95% CI: 0.98–0.99; *P* = 0.004), base excess (OR: 0.90; 95% CI: 0.87–0.93; *P*<0.001), hemoglobin (OR: 0.95; 95% CI: 0.95–0.96; *P*<0.001), displaced pelvic fracture (OR: 2.13; 95% CI: 1.48–3.06; *P*<0.001), and a positive computed tomography scan or positive focused assessment with sonography for trauma (OR: 1.62; 95% CI: 1.21–2.18; *P* = 0.001). Model 1, which was developed based on LASSO feature selection results and LR, displayed excellent discrimination (AUC: 0.894; 95% CI: 0.875–0.912), good calibration (*P* = 0.405), and clinical utility. In addition, the predictive power of model 1 was better than that of model 2 (AUC: 0.718; 95% CI: 0.679–0.757). Model 1 was deployed as a public web tool (http://82.156.217.249:8080/).

**Conclusions:**

Our study developed and validated prediction models to assist medical staff in the early diagnosis of massive hemorrhage in trauma. An open web calculator was developed to facilitate the practical application of the research results.

**Supplementary Information:**

The online version contains supplementary material available at 10.1186/s12873-022-00737-y.

## Background

Trauma is a major global public health problem. According to the World Health Organization, 5.8 million people die from trauma each year, accounting for 10% of all deaths. Trauma is also the leading cause of death for people under the age of 40 worldwide [1]. Massive hemorrhage is one of the most serious and life-threatening complications caused by trauma, and it is the main cause of preventable death in patients with trauma. About 40% of trauma deaths are attributed to massive hemorrhage [2–4]. Grossly visible massive hemorrhages can be treated in time by local compression, closure, and operation, and the survival rate of patients with trauma is higher in medical institutions. However, invisible hemorrhage may be overlooked by medical staff. Without intervention, the patients can develop sequential organ failure, coagulation dysfunction, and even death due to insufficient blood perfusion in a short time. If medical staff can identify the condition of traumatic massive hemorrhage early, intervene quickly, and actively adjust the treatment strategy, the disability and mortality rate due to massive traumatic hemorrhage may be reduced, and outcomes of severe trauma may be improved [5].

Presently, most prediction models related to traumatic hemorrhage are scoring systems based on traditional stepwise regression models [6–9], such as the trauma-associated severe hemorrhage (TASH) score [10] and the Prince of Wales (PWH) score [11]. However, these traditional scores are difficult to solve the problem of multicollinearity among a large number of potential predictive variables in feature selection. In addition, these scores usually require the manual calculation of results, which is time-consuming and complex. Lastly, the accuracy of these scores decreases by varying degrees over time and when applied to people in different regions [12].

Recently, machine learning has played an important role in the construction of various clinical prediction models. Least absolute shrinkage and selection operator (LASSO) regression is a powerful machine learning tool based on the bias-variance tradeoff theory of feature selection, which can minimize the potential collinearity of predictive variables and prevent over-fitting of prediction models [13]. However, little attention has been paid to its contribution to the field of traumatic hemorrhage prediction models. Moreover, vital sign indexes are objective measures of human physiological and pathological changes. Vital sign monitoring is non-invasive and presents the opportunity for early detection of massive hemorrhage in trauma. Therefore, this study intended to develop a logistic regression (LR) model for massive hemorrhage in trauma based on LASSO regression and compare it with an LR model based on vital sign indexes, as well as with clinically validated scores, such as the TASH and PWH scores. Finally, an open web calculator was developed to promote the convenient application of the model and assist doctors and nurses to identify traumatic massive hemorrhage in the early stage.

## Methods

### Data sources

The data of this study are from the trauma database of the General Hospital of the Chinese People’s Liberation Army (PLA) (hereinafter referred to as the trauma database). We included all patients with trauma in the trauma database who entered the emergency rescue room between January 2015 and March 2022, while excluding patients under the age of 16, those with second or further repeat admissions after trauma, and a data loss rate of more than 20%. The use of relevant de-identified data from the trauma database was reviewed by the Medical Ethics Committee of Chinese PLA General Hospital; the ethical batch number is S2021–466-01 and written informed consent was waived due to the study design and the harmless use of retrospective data.

### Potential predictive variables

A total of 62 potential predictive variables were included in this study, including demographic data, as well as the first recording of clinical symptoms, vital signs, laboratory tests, and imaging results after admission (Table 1). Among them, demographic data included sex and age. Clinical symptoms included unconsciousness and oliguria or anuria. Vital signs included heart rate (HR), respiratory rate (RR), pulse pressure (PP), body temperature, and peripheral oxygen saturation (SpO_2_). Laboratory tests included blood gas analysis, blood routine test, coagulation function, liver function, kidney function, electrolytes, myocardial enzymes, and other hematological tests. Imaging results included displaced pelvic fractures and results of computed tomography (CT) scan or focused assessment with sonography for trauma (FAST) positive for traumatic hemorrhage.

**Table 1:** Comparison of baseline characteristics of patients with trauma in trauma dataset

Characteristic	Total	Massive Hemorrhage		*P* value
		Yes	No	
No.	2353	377	1976	
Male, n (%)	1819 (77.31%)	273 (72.41%)	1546 (78.24%)	0.013*
Age, mean (SD), y	47.25 (17.34)	46.48 (16.01)	47.40 (17.59)	0.340
Clinical symptoms, n (%)				
Unconsciousness	522 (34.30%)	95 (41.67%)	427 (33.00%)	0.011*
Oliguria or anuria	108 (5.54%)	19 (6.27%)	89 (5.41%)	0.550
Vital signs, mean (SD)				
Heart rate, beats/min	95.45 (24.22)	108.33 (27.15)	92.98 (22.81)	< 0.001*
Respiratory rate, breaths/min	20.13 (2.40)	20.17 (2.23)	19.91 (3.14)	0.051
Pulse pressure, mmHg	50.50 (17.58)	43.72 (16.03)	51.78 (17.57)	< 0.001*
Body temperature, °C	36.75 (0.77)	36.63 (0.87)	36.77 (0.75)	0.001*
Peripheral oxygen saturation, %	96.25 (5.08)	94.86 (7.78)	96.52 (4.34)	< 0.001*
Laboratory findings, mean (SD)				
Pondus Hydrogenii	7.39 (0.09)	7.35 (0.13)	7.40 (0.08)	< 0.001*
PaO_2_, mmHg	123.01 (67.01)	131.89 (83.60)	121.29 (63.19)	0.006*
PaCO_2_, mmHg	36.84 (8.76)	35.05 (11.71)	37.18 (8.03)	< 0.001*
Lactic acid, mmol/L	2.89 (2.73)	4.53 (3.94)	2.57 (2.31)	< 0.001*
Base excess, mmol/L	−2.13 (4.80)	−5.49 (5.97)	−1.48 (4.25)	< 0.001*
Hemoglobin, g/L	123.26 (28.50)	91.22 (28.32)	129.38 (24.11)	< 0.001*
Mean corpuscular volume, fl	88.86 (5.17)	88.72 (6.45)	88.89 (4.89)	0.560
Mean Corpuscular Hemoglobin, pg	30.97 (2.10)	30.76 (2.87)	31.01 (1.92)	0.037*
MCHC, g/L	348.48 (13.71)	346.34 (16.69)	348.89 (13.02)	< 0.001*
RDW, %	12.84 (1.30)	13.28 (1.78)	12.76 (1.17)	< 0.001*
Platelet count, × 10^9^/L	202.42 (82.15)	169.67 (78.80)	208.70 (81.30)	< 0.001*
Mean platelet volume, fl	10.37 (1.02)	10.45 (1.04)	10.35 (1.01)	0.090
WBC count, ×10^9^/L	14.73 (6.50)	15.66 (7.35)	14.55 (6.31)	0.002*
Neutrophil count, ×10^9^/L	0.88 (1.07)	1.02 (2.60)	0.85 (0.23)	0.004*
Eosinophil count, × 10^9^/L	0.00 (0.02)	0.00 (0.05)	0.00 (0.01)	0.340
Basophil count, ×10^9^/L	0.00 (0.00)	0.00 (0.00)	0.00 (0.00)	< 0.001*
Lymphocyte count, ×10^9^/L	0.12 (0.35)	0.17 (0.85)	0.10 (0.10)	< 0.001*
Monocyte count, ×10^9^/L	0.05 (0.05)	0.06 (0.11)	0.05 (0.02)	0.002*
Fibrinogen, g/L	2.92 (1.66)	2.30 (1.85)	3.04 (1.59)	< 0.001*
Thrombin time, s	17.24 (14.30)	18.71 (16.12)	16.96 (13.92)	0.030*
Prothrombin Time, s	15.89 (8.30)	19.76 (13.66)	15.16 (6.58)	< 0.001*
APTT, s	37.55 (16.07)	45.11 (25.14)	36.12 (13.23)	< 0.001*
International normalized ratio	1.27 (0.65)	1.68 (1.35)	1.19 (0.33)	< 0.001*
D-dimer level, μg/mL	9.93 (7.73)	12.85 (7.61)	9.37 (7.64)	< 0.001*
Alanine aminotransferase, U/L	98.57 (287.07)	139.51 (341.30)	90.75 (274.91)	0.002*
Aspartate aminotransferase, U/L	133.15 (532.54)	198.93 (521.61)	120.59 (533.81)	0.009*
γ-glutamyl transferase, U/L	47.03 (79.39)	39.31 (56.80)	48.50 (82.94)	0.039*
Alkaline phosphatase, U/L	71.16 (52.89)	62.53 (68.06)	72.81 (49.31)	< 0.001*
Total bilirubin, umol/L	16.85 (28.78)	14.64 (30.66)	17.27 (28.40)	0.100
Direct bilirubin, umol/L	7.13 (21.74)	6.81 (23.86)	7.20 (21.32)	0.750
Total protein, g/L	62.38 (11.01)	49.99 (12.04)	64.75 (9.05)	< 0.001*
Albumin, g/L	37.45 (7.35)	29.92 (7.66)	38.89 (6.34)	< 0.001*
Blood creatinine, umol/L	87.64 (74.45)	108.37 (104.94)	83.68 (66.38)	< 0.001*
Blood urea nitrogen, mmol/L	6.83 (4.87)	7.84 (6.33)	6.64 (4.51)	< 0.001*
Serum uric acid, umol/L	341.32 (140.79)	354.63 (147.54)	338.78 (139.36)	0.045*
Sodium, mmol/L	139.20 (5.01)	140.28 (4.96)	139.00 (5.00)	< 0.001*
Potassium, mmol/L	3.83 (0.61)	3.90 (0.77)	3.82 (0.58)	0.013*
Calcium, mmol/L	1.91 (0.41)	1.80 (0.33)	1.93 (0.42)	< 0.001*
Chloride, mmol/L	104.60 (5.69)	107.58 (6.41)	104.03 (5.36)	< 0.001*
Phosphorus, mmol/L	0.97 (0.43)	1.13 (0.60)	0.94 (0.37)	< 0.001*
Magnesium, mmol/L	0.81 (0.13)	0.79 (0.15)	0.82 (0.12)	0.003*
Creatine kinase, U/L	1179.65 (4600.74)	1791.84 (4063.52)	1063.05 (4687.99)	0.005*
Creatine kinase isoenzyme, ng/ml	12.18 (31.01)	20.73 (33.47)	10.54 (30.26)	< 0.001*
Myoglobin, ng/ml	914.82 (2677.44)	1957.96 (3744.09)	715.80 (2370.86)	< 0.001*
Troponin T, ng/ml	0.10 (1.23)	0.16 (0.34)	0.09 (1.34)	0.330
Pro-BNP, pg/ml	627.56 (3075.27)	690.54 (3209.17)	615.57 (3049.79)	0.660
Amylase, U/L	103.47 (196.55)	135.41 (230.84)	97.38 (188.76)	< 0.001*
Lipase, U/L	143.74 (366.58)	166.30 (336.37)	139.44 (371.98)	0.190
Lactate dehydrogenase, U/L	415.85 (514.39)	503.69 (510.98)	399.12 (513.46)	< 0.001*
Blood glucose, mmol/L	9.08 (3.83)	10.45 (4.94)	8.82 (3.52)	< 0.001*
C-reactive protein, mg/dl	2.47 (4.78)	2.52 (4.65)	2.46 (4.80)	0.810
Imaging results, n (%)				
Displaced pelvic fracture	272 (11.56%)	96 (25.46%)	176 (8.91%)	< 0.001*
CT scan or FAST positive	883 (37.53%)	216 (57.30%)	667 (33.76%)	< 0.001*

*P* value is the difference analysis result between the massive hemorrhage group and the non-massive hemorrhage group. The quantitative data were expressed by mean (SD); the differences were analyzed by the *t*-test. The classified data were expressed by n (%), and the differences were analyzed by the Chi-square test. * represents the difference is statistically significant. *SD* Standard deviation, *PaO*_*2*_ Partial pressure of oxygen in arterial blood, *PaCO*_*2*_ partial pressure of carbon dioxide in arterial blood, *MCHC* Mean corpuscular hemoglobin concentration, *RDW* Red cell distribution width, *WBC* White blood cell, *APTT* Activated partial thromboplastin time, *Pro-BNP* Pro-brain natriuretic peptide, *CT* Computed tomography; *FAST* focused assessment with sonography for trauma

### Outcomes

In this study, massive hemorrhage was used as the outcome variable of the prediction model. Presently, there is no authoritative standard for the definition of massive hemorrhage. By consulting previous literature and combining our findings with clinical practice, we determined the screening criteria as follows [4, 14, 15]: 1) massive transfusion of three or more units of red blood cells (RBC) within 1 hour at any time during the first 24 hours after admission; 2) embolization or hemostatic surgery within 24 hours after admission. If the patients met either of the mentioned conditions, they were classified into the massive hemorrhage group; otherwise, they were classified into the non-massive hemorrhage group.

### Variable selection and model construction

In this study, two models for predicting massive hemorrhage in trauma were developed, and the accuracies of the models were compared. First of all, the feature selection was carried out based on LASSO regression [16], and the variable coefficients of 62 potential predictive variables were punished by the penalty coefficient λ so that the coefficients of relatively unimportant variables became 0; these variables were then excluded from the model. We selected the predictive variables that were shown to have a significant influence on the outcome. The value of λ was determined by ten-fold cross-validation, and the most predictable feature combination was selected by the 1 standard error of the minimum criteria (the 1-se criteria). The LR model was subsequently developed according to the feature combination (hereinafter referred to as model 1). Secondly, based on the first vital sign data of the patients after admission, including HR, RR, PP, body temperature, and SpO_2_, the LR model (hereinafter referred to as model 2) was developed. In this study, model 1 and model 2 both used ten-fold cross-validation. Models 1 and 2 were compared with the commonly used clinical scores related to traumatic hemorrhage: the TASH and PWH scores. Finally, the corresponding web calculator was developed based on the model with the best prediction effect (Supplementary Fig. 1).

### Model evaluation and statistical analysis

The discrimination of the prediction model was assessed using the receiver operating characteristic (ROC) curve, and the calibration plot together with the unreliability test was used for evaluating the accuracy of the prediction [17]. The clinical utility of the model was evaluated with decision curve analysis [18]. We used Stata17, R4.1.3 for statistical analysis, and Java. JDK1.8 for web calculator development. The quantitative data were expressed as mean (standard deviation), the differences were analyzed by the *t*-test; the classified data were expressed by n (%), and the differences were analyzed by the Chi-square test. *P* < 0.05 was considered statistically significant.

## Results

### Comparison of baseline characteristics

**Fig. 1:**
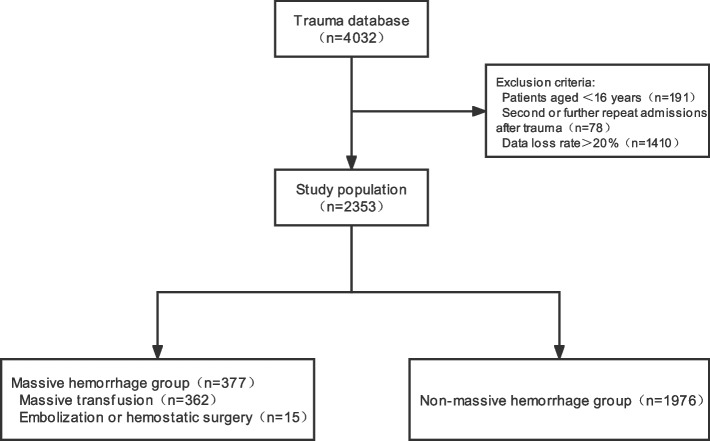
Flow chart of patient selection from the trauma database

This study included 4032 patients with trauma in the emergency department rescue room in the trauma database. After applying the exclusion criteria, 2353 patients remained, with an average age (standard deviation) of 47.25 (17.34) years; 1819 (77.31%) patients were male. Among them, there were 377 (16.02%) patients with massive hemorrhage, including 362 (15.38%) patients who underwent massive transfusion and 15 (0.64%) who underwent embolization or hemostatic surgery (Fig. 1). We extracted 62 potential predictive variables for each patient and compared the baseline characteristics of the patients in the massive hemorrhage group with those of the patients in the non-massive hemorrhage group (Table 1). According to statistical analysis, the patients with massive hemorrhage were more likely to have unconsciousness (*P* = 0.011), accelerated HR (*P*<0.001), decreased PP (*P*<0.001) and SpO_2_ (*P*<0.001), decreased base excess (BE) (*P*<0.001) and hemoglobin (HB) (*P*<0.001), displaced pelvic fracture (*P*<0.001), and a CT scan or FAST positive for hemorrhage (*P*<0.001).

### Development of a prediction model of massive hemorrhage in trauma based on LASSO feature selection

We included 62 potential predictive variables commonly measured at admission in LASSO regression for feature selection, used ten-fold cross-validation to determine the penalty coefficient λ, and took 1 standard error (1-se) of the minimum λ as the optimal value (Supplementary Fig. 2a, 2b). The final feature combination contained seven indicators, namely: HR, PP, HB, BE, C-reactive protein, displaced pelvic fracture, and a positive CT scan or FAST. C-reactive protein was excluded because there was no significant difference in univariate analysis (*P* = 0.810). Finally, based on HR, PP, HB, BE, displaced pelvic fracture, and positivity of CT scan or FAST, we developed a multivariable LR model, namely, model 1. The OR (95% CI) and *P* values of 6 predictive variables in model 1 are shown in Table 2.

**Table 2:** Multivariable logistic regression model based on LASSO feature selection

Intercept and Variables	β	OR (95% CI)	*P* value
Heart rate	0.013	1.01 (1.01, 1.02)	< 0.001
Pulse pressure	−0.014	0.99 (0.98, 0.99)	0.004
Base excess	−0.104	0.90 (0.87, 0.93)	< 0.001
Hemoglobin	−0.048	0.95 (0.95, 0.96)	< 0.001
Displaced pelvic fracture	0.757	2.13 (1.48, 3.06)	< 0.001
CT scan or FAST positive	0.484	1.62 (1.21, 2.18)	0.001
Intercept	2.419		< 0.001

*LASSO* Least absolute shrinkage and selection operator, *OR* Odds ratio, *CI* Confidence interval, *CT* Computed tomography, *FAST* Focused assessment with sonography for trauma

**Fig. 2:**
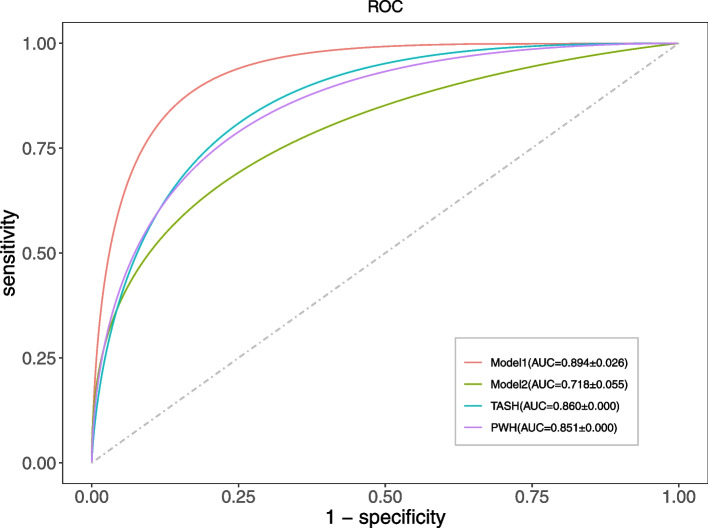
Comparison of ROC curves of model 1, model 2, TASH score, and PWH score. ROC: receiver operating characteristic curve; AUC: area under the curve; TASH: trauma-associated severe hemorrhage; PWH: Prince of Wales

**Fig. 3:**
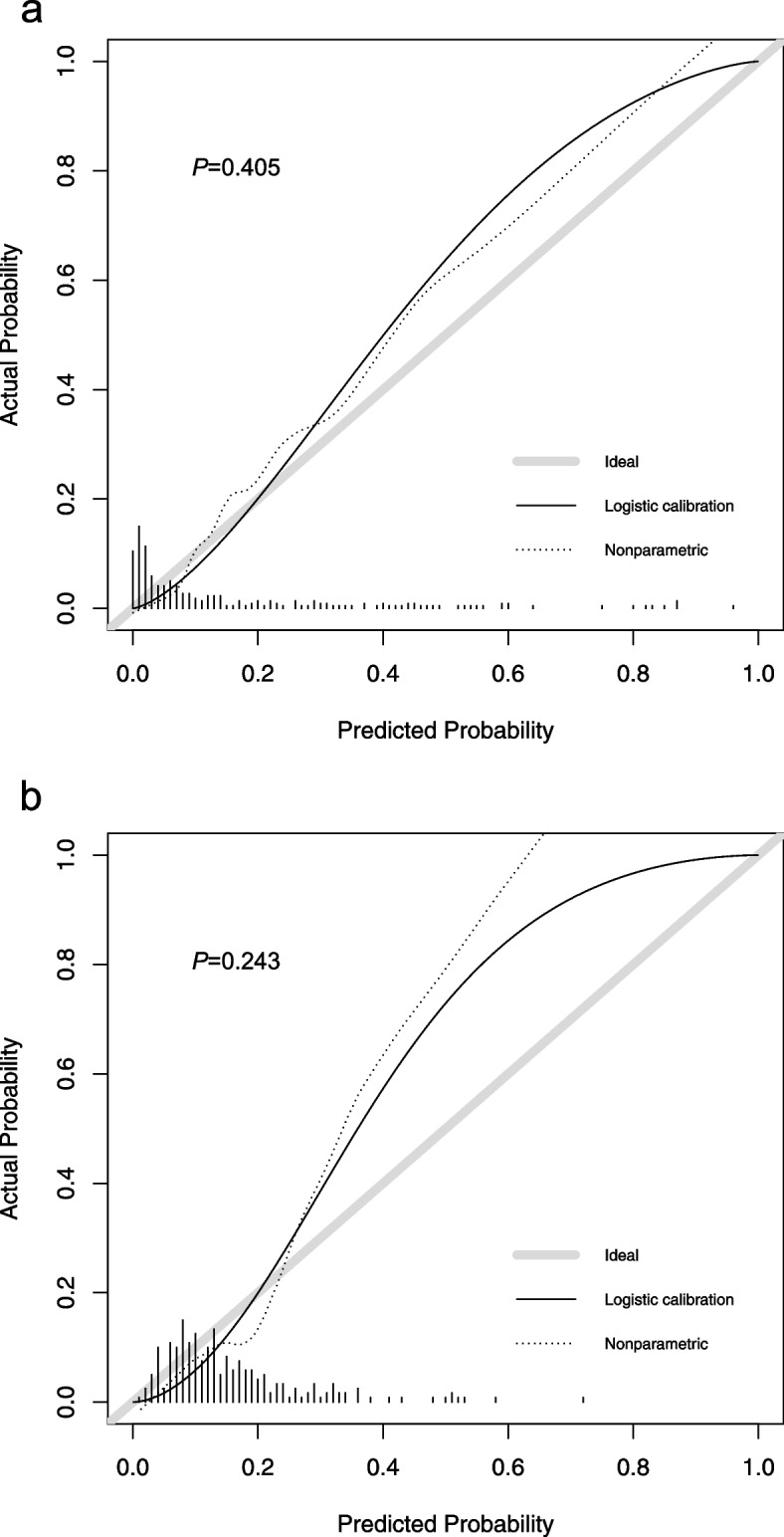
The calibration plot of the models for predicting massive hemorrhage in trauma. a: model 1 (*P* = 0.405); b: model 2 (*P* = 0.243). Calibration focuses on the accuracy of the absolute risk prediction of the model, that is, the consistency between the probability of massive hemorrhage in trauma predicted by the model and that actually observed. The y-axis represents the actual rate of massive hemorrhage. The x-axis represents the predicted probability of massive hemorrhage. For a well-calibrated model, the scatter points should be arranged along a 45-degree diagonal line. *P* > 0.05 means no significant difference, and the calibration of the model is good

**Fig. 4:**
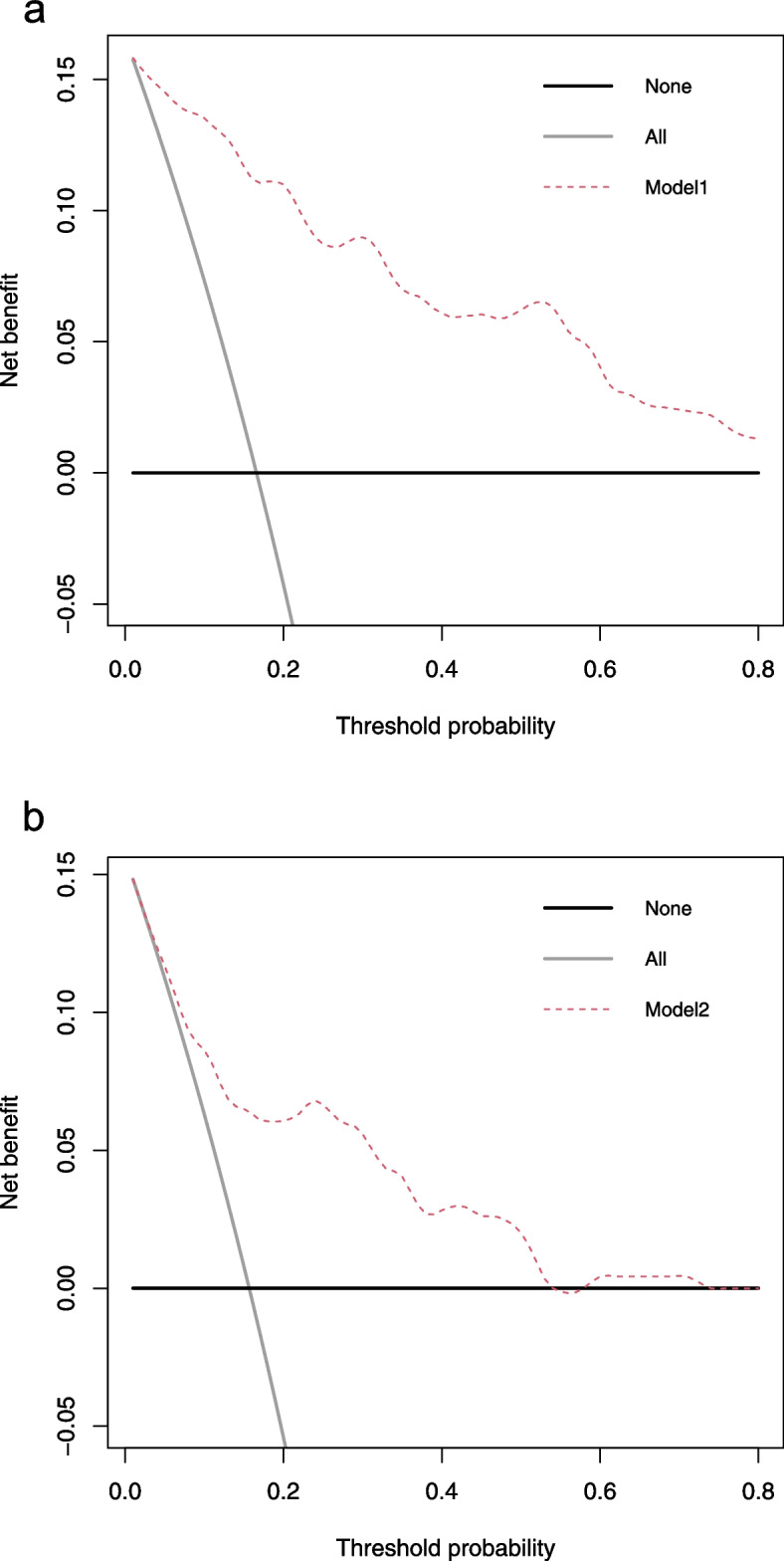
Decision curve analysis of the models for predicting massive hemorrhage in trauma. a: model 1; b: model 2. The y-axis measures the net benefit. The x-axis represents the threshold probability. The red line represents the model’s decision curve. The “All” line represents the assumption that all patients have a massive hemorrhage. The “None” line represents the assumption that no patients have a massive hemorrhage. The further the model’s decision curve is from the “All” and “None” reference lines, the higher the net benefit of the model at the same threshold probability

We used ten-fold cross-validation for the internal validation of model 1. First of all, by drawing the ROC curve of model 1 (Fig. 2), the area under the curve (AUC) was calculated to be 0.894 (95% CI: 0.875–0.912), which shows that model 1 has excellent discrimination and can adequately distinguish between trauma patients with and without massive hemorrhage. Secondly, the calibration curve of model 1 (Fig. 3a) shows that model 1 has a good calibration (*P* = 0.405) and that the prediction risk of massive hemorrhage in trauma is close to its actual risk. Finally, the decision curve of model 1 (Fig. 4a) shows that the use of model 1 to predict the risk of massive hemorrhage adds more clinical value than the assumption that all patients have massive hemorrhage or non-massive hemorrhage.

### Development of a prediction model of massive hemorrhage in trauma based on vital signs

We further used vital signs as predictive variables and developed model 2 based on the LR method. The OR (95% CI) and *P* values of the five predictive variables of model 2 are shown in Table 3. We used ten-fold cross-validation for internal validation of model 2. First of all, by drawing the ROC curve of model 2 (Fig. 2), the AUC was calculated to be 0.718 (95% CI: 0.679–0.757), which shows that model 2 has good discrimination. Secondly, the calibration curve of model 2 (Fig. 3b) showed that model 2 has a good calibration (*P* = 0.243). Finally, the decision curve of model 2 (Fig. 4b) shows that there are some clinical benefits from using model 2 to predict the risk of massive hemorrhage.

**Table 3:** Multivariable logistic regression model based on vital signs

Intercept and Variables	β	OR (95% CI)	*P* value
Heart rate	0.023	1.02 (1.02, 1.03)	< 0.001
Pulse pressure	−0.025	0.98 (0.97, 0.98)	< 0.001
Respiratory rate	−0.058	0.94 (0.90, 0.99)	0.017
Body temperature	−0.264	0.77 (0.65, 0.90)	< 0.001
Peripheral oxygen saturation	−0.026	0.97 (0.96, 0.99)	0.010
Intercept	10.553		< 0.001

*OR* Odds ratio, *CI* Confidence interval

### Comparison of prediction models for massive hemorrhage in trauma

We compared the effects of model 1 and model 2. First, the AUCs of models 1 and 2 were found to be significantly different (*P* < 0.001). The discrimination of model 1 was better than that of model 2. Secondly, the calibration curve of model 1 (*P* = 0.405) was closer to the diagonal line of the calibration plot than that of model 2 (*P* = 0.243), with better calibration (Fig. 3a, b). Finally, the decision curve of model 1 was further away from the two reference lines of “All” and “None” and had a higher net benefit than model 2 under the same threshold probability (Fig. 4a, b). In general, model 1, based on LASSO feature selection, has a better performance than model 2, which was based solely on vital signs as predictive variables.

In addition, by comparing model 1 and model 2 with TASH score (AUC: 0.860) and PWH score (AUC: 0.851) (Supplementary Table 1), we found that the AUC of model 1 was better than those of the TASH and PWH scores; the AUCs of the TASH and PWH scores were better than that of model 2 (Fig. 2).

### Development of web calculator for prediction model of massive hemorrhage in trauma

To facilitate the use and validation of our model by medical staff and the public at home and abroad, we have developed a public web calculator (Supplementary Fig. 3) for the traumatic massive hemorrhage prediction model based on LASSO feature selection (model 1). The website is http://82.156.217.249:8080/; it supports the clinical early prediction of massive hemorrhage risk in trauma.

## Discussion

We developed two clinical prediction models, then launched a public web calculator based on the better model, so that healthcare workers, as well as the public around the world, can use and validate our model. Clinicians can use the web calculator to personalize the risk of massive hemorrhage in hospitalized patients with trauma. If the estimated risk of massive hemorrhage in a patient is low, clinicians may choose to continue monitoring the patient, while high-risk estimates may be more supportive of aggressive massive transfusion protocols or surgical intervention.

LASSO regression is a feature selection method that has attracted much attention and has been widely used in recent years. It introduces L1 regularization based on least squares regression, which can negate a certain degree of overfitting and improve the predictive performance of the model for unknown samples. LASSO is different from traditional stepwise regression, as it can deal with all variables at the same time, not in a stepwise manner. If the correlation between potential predictive variables is high, LASSO chooses only one of them and reduces the coefficients of other predictive variables to 0, so it can solve the problem of multicollinearity between variables. In addition, LASSO has many advantages, such as a fast calculation speed and easy model interpretation, among others [16]. Model 1, which was developed based on LASSO regression, was preliminarily proven to be better than the TASH score, PWH score, and model 2.

Presently, the prediction models related to traumatic hemorrhage reported in the literature mainly use massive transfusion as the outcome variable [19–22]. However, massive transfusion cannot explain all the clinically important results related to massive hemorrhage. For example, before the standard for massive transfusion is reached, hemostatic intervention may have been performed for patients with massive hemorrhage. Therefore, competing risk bias may occur when massive transfusion is used alone as an outcome variable in a prediction model [15]. In addition, the traditional definition of massive transfusion of ≥10 units of RBCs within 24 hours should be considered obsolete. The critical administration threshold (CAT) that defines the reception of three or more units of RBCs during a single hour anytime during the first 24 hours of arrival can identify patients with massive hemorrhage more accurately and minimize survivor bias [4, 23]. Therefore, our study used that definition of massive transfusion or “embolization or hemostatic surgery” within 24 hours of admission as the screening criteria for traumatic massive hemorrhage, emphasizing the severity of hemorrhage rather than the amount of blood transfusion, and using the modern definition of CAT instead of the traditional definition of massive transfusion.

In this study, model 1 was developed based on HR, PP, BE, HB, displaced pelvic fracture, and a positive CT scan or FAST. In clinical practice, the above six indices play an important role in indicating the condition of traumatic massive hemorrhage. Previous studies have also found that the above indicators are closely related to traumatic hemorrhage. For example, the traumatic bleeding severity score based on the clinical data of 119 patients with severe trauma included systolic blood pressure (SBP), a positive FAST, and pelvic fractures as predictive variables [24]. The Vandromme score, based on the data of 6638 trauma patients from the Trauma Center of the University of Alabama at Birmingham, included HR, SBP, and HB as predictive variables [25]. The Larson score, developed based on the data of US military service personnel in the Joint Theater Trauma Registry transfusion database, included HR, SBP, base deficit, and HB as predictive variables [26].

Among the current scoring systems related to traumatic massive hemorrhage, we selected the widely employed TASH and PWH scores to compare with our clinical prediction model. The TASH score was originally developed and validated based on the data of 6044 patients with severe blunt trauma in the TraumaRegister DGU® database [10]. In the original study with the TASH score, AUC was 0.887 (95% CI: 0.864–0.910). The PWH score was developed and validated based on the data of 1891 trauma patients in the administrative trauma database of the Prince of Wales Hospital [11]. In the original study of the PWH score, the sensitivity was 31.5%, the specificity was 99.7%, and the AUC was 0.889. The TASH and PWH scores were externally validated in the trauma dataset and compared with model 1 (Fig. 2). The predictive ability of model 1 was better than that of the TASH score as well as that of the PWH score. Thus, the TASH and PWH scores were found to maintain a high AUC in the original literature research, but, when applied to the trauma dataset of Chinese PLA General Hospital for external validation, the AUC decreased slightly, and the respective accuracies of the two models were not as good as that of model 1. This may be caused by the differences in physiology, disease processes, and disease outcomes of the study population caused by regional and ethnic differences [27]. In addition, with the passage of time, the effectiveness of the models will decline variably; parameters need to be continuously adjusted and optimized.

This study further developed model 2 based on the first recorded data of five vital sign indicators after admission. The reasons for choosing vital signs indicators to develop a prediction model include: first of all, since vital sign data are easy to obtain in both pre-hospital and in-hospital environments, medical staff can easily record the data regularly and make risk predictions the first time after admission, instead of waiting for laboratory test results and imaging results, which helps to improve the timeliness of the model. Second, simple feature selection ensures that the prediction model can be recalculated based on repeatedly measured vital sign data during pre-hospital first aid or emergency triage, providing valuable information about whether the patients are responding to treatment. This makes it easier for medical professionals to modify their treatment plans. Finally, the simplicity of the prediction model input and output improves the interpretability of the model, which increases the likelihood that healthcare providers will trust the model’s predicted results. In this study, the predictive ability of model 2 was not as high as that of model 1, and there was a certain gap between model 2 and traditional TASH and PWH scores as well. However, thanks to the advantages of immediate prediction, repeatable prediction, and timeliness, model 2 has good clinical practical value and is still worthy of further exploration in the future.

This study had certain limitations. First, the study population comprised adult patients, and further subgroup study based on age was not considered. Age plays an important role in predicting the risk of traumatic massive hemorrhage. Some studies have shown that the physiological reserve of elderly patients is less than that of their younger counterparts and that they are more likely to have massive hemorrhages [28]. In future research, we will divide the patients into different subgroups according to their age for further analysis. Second, the study used two screening strategies including massive transfusion and embolization or hemostatic surgery to cover as many trauma patients with massive hemorrhage as possible. It is undeniable that a small number of patients were still not included in the study population because they either refused to undertake the examination and treatment or died before receiving treatment. We will include trauma patients with massive hemorrhage-related deaths in future studies to further reduce survivor bias. Third, the prediction models of massive hemorrhage in trauma can only guide the doctor’s clinical decision-making process and cannot replace the doctor’s clinical judgment and other diagnostic tests. Finally, this was a retrospective observational study. Although the quality of the trauma database is high, there are still data losses and input errors. In future studies, we will include multicenter data, further expand the sample size, conduct external validation, include prospectively collected data, and further validate and evaluate the effectiveness of the traumatic prediction models in clinical practice.

## Conclusions

We developed two clinical prediction models and a corresponding web calculator. Among them, the multivariable LR model developed based on LASSO feature selection results, which includes six variables commonly measured on admission to a hospital, has better prediction effects than the multivariable LR model developed based on vital signs and common clinical scores related to traumatic hemorrhage. Estimating the risk of massive hemorrhage in trauma could help identify trauma patients who are and are not likely to develop massive hemorrhage to ensure appropriate treatment and optimize the use of medical resources.

## Supplementary Information

**Additional file 1: Supplementary Fig. 1.** Flow chart of variable selection and model construction. LASSO: least absolute shrinkage and selection operator; LR: logistic regression.

**Additional file 2: Supplementary Fig. 2.** Feature selection using LASSO regression. a: Identification of the optimal penalization coefficient lambda (λ) in the LASSO used ten-fold cross-validation and the 1 standard error of the minimum criteria (the 1-se criteria). b: LASSO coefficient profiles of the features. A vertical line is drawn at the selected optimal λ, and the corresponding indexes of the curve intersecting the vertical line are the selected characteristic indexes. LASSO: least absolute shrinkage and selection operator.

**Additional file 3: Supplementary Table 1.** TASH score and PWH score parameters.

**Additional file 4: Supplementary Fig. 3.** The web calculator for the prediction model of massive hemorrhage in trauma. Input the test results of heart rate, pulse pressure, base excess and hemoglobin, and select whether the patient has displaced pelvic fracture and positive CT scan or FAST. Then click the “CALCULATE” button, and the web calculator will automatically calculate and obtain the risk probability of massive hemorrhage. The range is 1–100%. The higher the value, the greater the risk of massive hemorrhage. CT: computed tomography; FAST: focused assessment with sonography for trauma.

## Data Availability

The trauma dataset is available from the trauma database of Chinese PLA General Hospital, although restrictions apply to the availability of these data, which were used under license for the current study, and so are not publicly available. Data are however available from the authors upon reasonable request and with permission of the Chinese PLA General Hospital.

## Abbreviations

LR: Logistic regression
LASSO: Least absolute shrinkage and selection operator
TASH: Trauma-associated severe hemorrhage
PWH: Prince of Wales
HR: Heart rate
RR: Respiratory rate
PP: Pulse pressure
SpO_2_: Peripheral oxygen saturation
CT: Computed tomography
FAST: Focused assessment with sonography for trauma
RBC: Red blood cell
ROC: Receiver operating characteristic curve
BE: Base excess
HB: Hemoglobin
AUC: Area under the curve
CAT: Critical administration threshold
SBP: Systolic blood pressure
