# Supplementary figures and images for: A prediction model for massive hemorrhage in trauma: a retrospective observational study
Source: BMC Emerg Med. 2022 Nov 14;22:180. doi: 10.1186/s12873-022-00737-y (PMC9661746; doi:10.1186/s12873-022-00737-y)

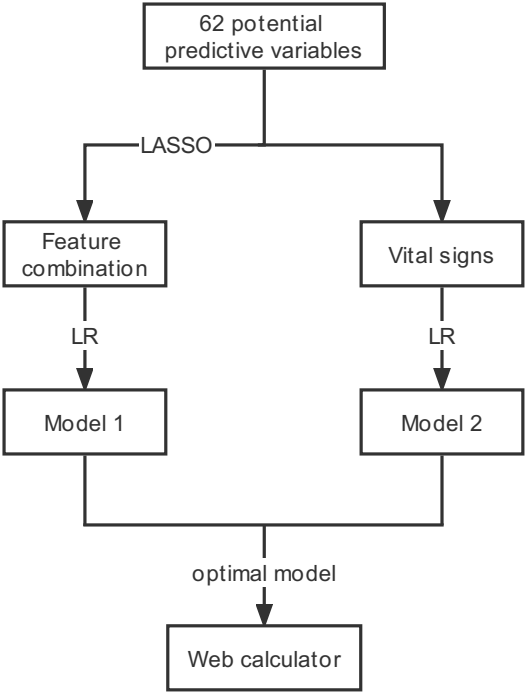

Supplement: Supplementary file 1 — Additional file 1: Supplementary Fig. 1. Flow chart of variable selection and model construction. LASSO: least absolute shrinkage and selection operator; LR: logistic regression. [file 12873_2022_737_MOESM1_ESM.pdf]

a

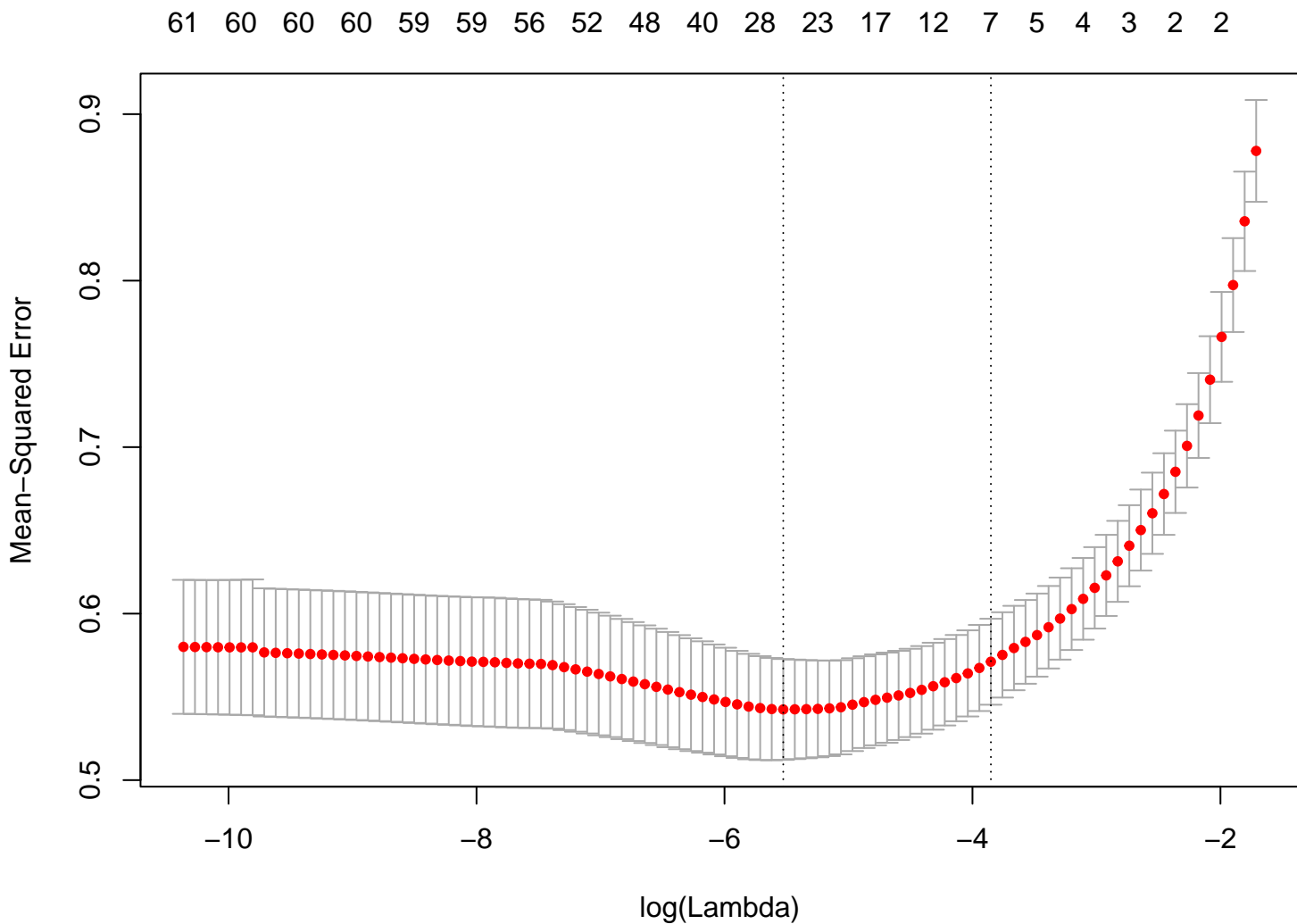

b

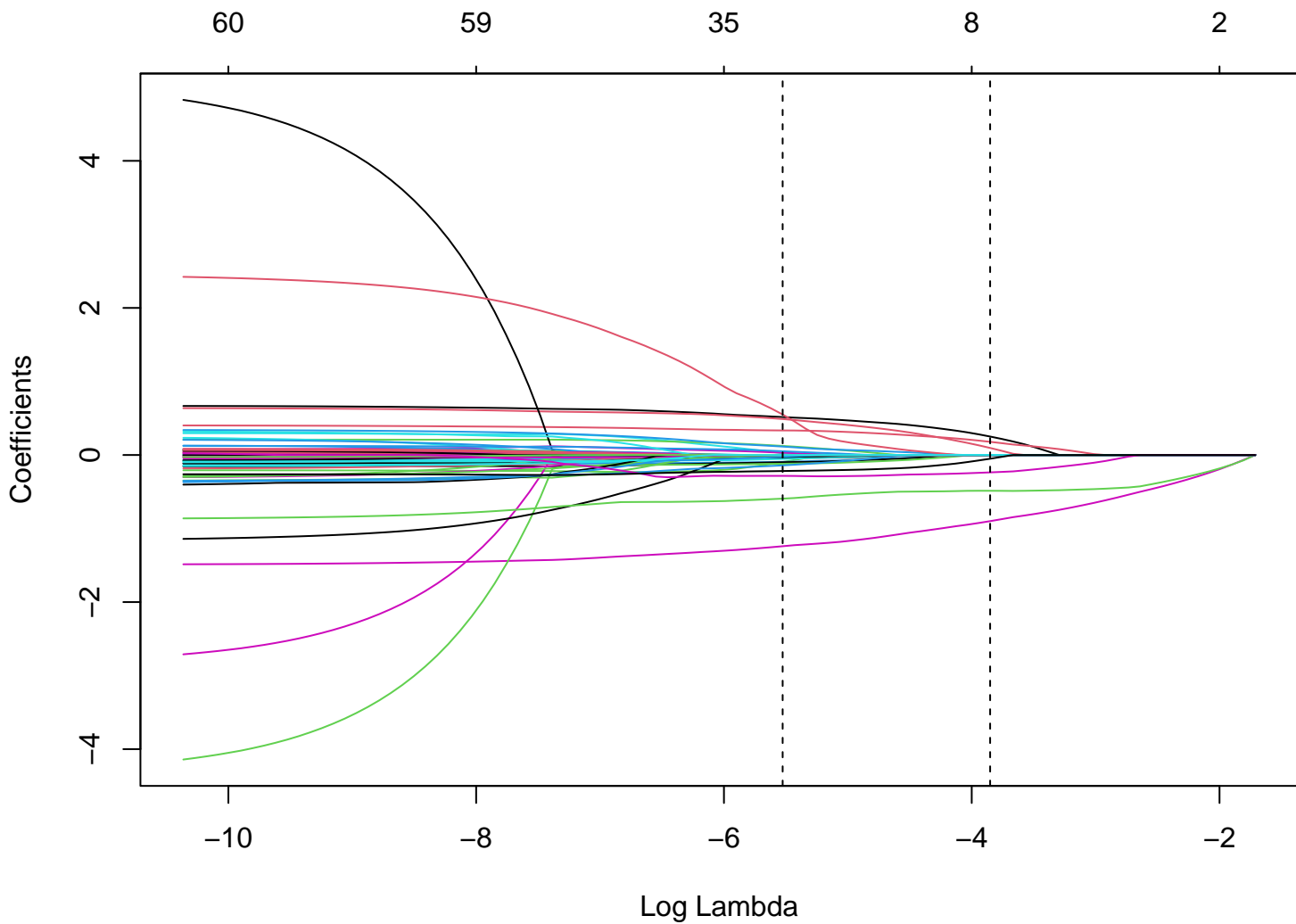

Supplement: Supplementary file 2 — Additional file 2: Supplementary Fig. 2. Feature selection using LASSO regression. a: Identification of the optimal penalization coefficient lambda (λ) in the LASSO used ten-fold cross-validation and the 1 standard error of the minimum criteria (the 1-se criteria). b: LASSO coefficient profiles of the features. A vertical line is drawn at the selected optimal λ, and the corresponding indexes of the curve intersecting the vertical line are the selected characteristic indexes. LASSO: least absolute shrinkage and selection operator. [file 12873_2022_737_MOESM2_ESM.pdf]

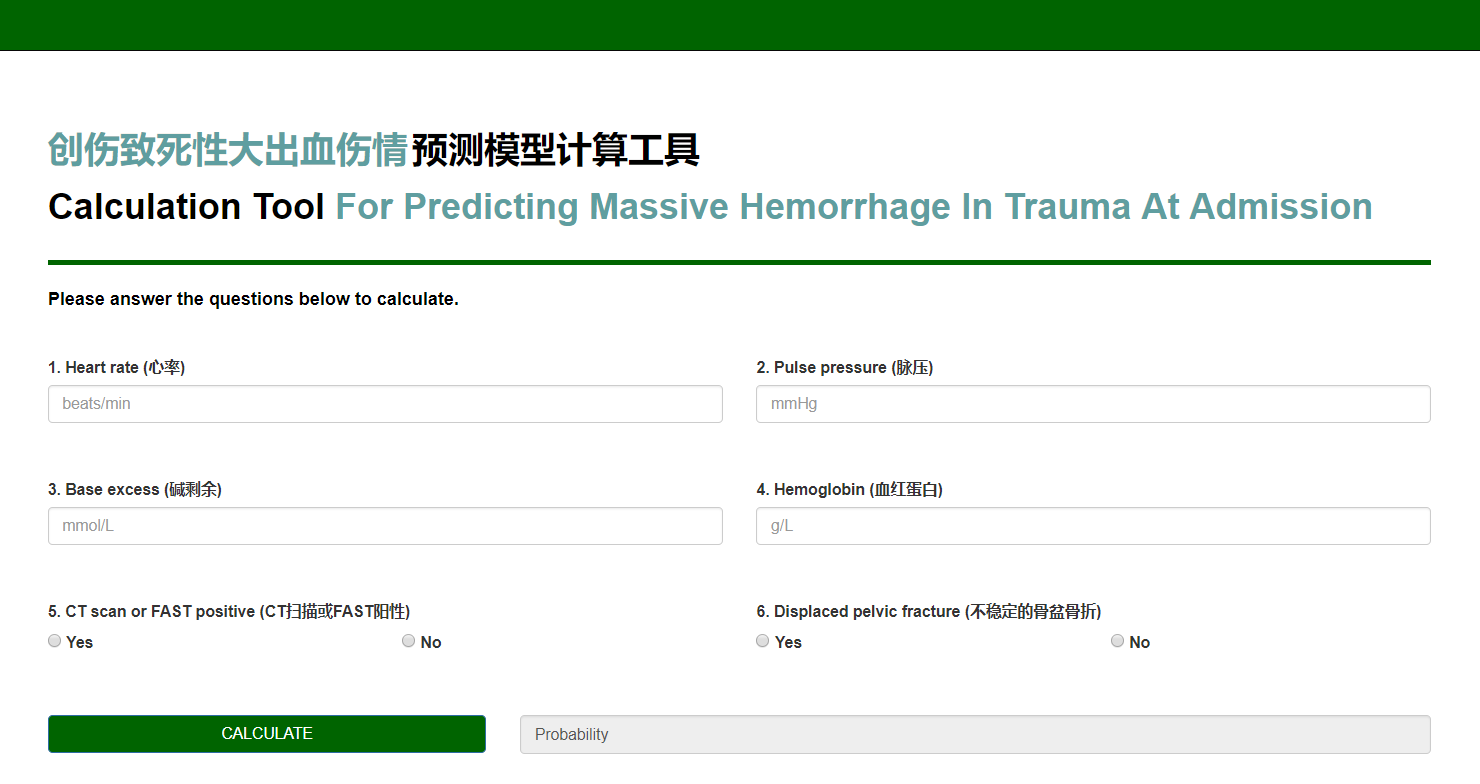

Supplement: Supplementary file 4 — Additional file 4: Supplementary Fig. 3. The web calculator for the prediction model of massive hemorrhage in trauma. Input the test results of heart rate, pulse pressure, base excess and hemoglobin, and select whether the patient has displaced pelvic fracture and positive CT scan or FAST. Then click the “CALCULATE” button, and the web calculator will automatically calculate and obtain the risk probability of massive hemorrhage. The range is 1–100%. The higher the value, the greater the risk of massive hemorrhage. CT: computed tomography; FAST: focused assessment with sonography for trauma. [file 12873_2022_737_MOESM4_ESM.png]
