# Supplementary material for: A prediction model for massive hemorrhage in trauma: a retrospective observational study
Source: BMC Emerg Med. 2022 Nov 14;22:180. doi: 10.1186/s12873-022-00737-y (PMC9661746; doi:10.1186/s12873-022-00737-y)
Supplement: Supplementary file 3 — Additional file 3: Supplementary Table 1. TASH score and PWH score parameters. [file 12873_2022_737_MOESM3_ESM.docx]

Supplementary table 1. TASH score and PWH score parameters

| Variables | TASH | |  | PWH | |
| --- | --- | --- | --- | --- | --- |
|  | Value | Pts |  | Value | Pts |
| Gender | Male | 1 |  |  |  |
| Heart rate(beats/min) | ＞120 | 2 |  | ≥120 | 1 |
| Systolic blood pressure(mmHg) | ＜100 | 4 |  | ≤90 | 3 |
|  | ＜120 | 1 |  |  |  |
| Base excess(mmol/L) | ＜-10 | 4 |  | BD＞5 | 1 |
|  | ＜-6 | 3 |  |  |  |
|  | ＜-2 | 1 |  |  |  |
| Hemoglobin(g/dl) | ＜7 | 8 |  | ≤7 | 10 |
|  | ＜9 | 6 |  | 7.1 to 10 | 1 |
|  | ＜10 | 4 |  |  |  |
|  | ＜11 | 3 |  |  |  |
|  | ＜12 | 2 |  |  |  |
| FAST | Positive | 3 |  | Or CT-positive | 2 |
| Pelvic fracture | Clinically unstable | 6 |  | Displaced | 1 |
| Femur fracture | Open and\or dislocated | 3 |  |  |  |
| GCS |  |  |  | ≤8 | 1 |

TASH: trauma-associated severe hemorrhage; PWH: Prince of Wales; BD: base deficit; FAST: focused assessment with sonography for trauma; CT: computed tomography; GCS: glasgow coma scale.
